# Sixth Annual Meeting of the American Medical Association, Held at New York, May, 1853

**Published:** 1853-08

**Authors:** 


### BUFFALO MEDICAL JOURNAL

AND

### MONTHLY REVIEW.

**VOL. 9.** AUGUST, 1853. **NO. 3.**

### ORIGINAL COMMUNICATIONS.

ART. I.—*Sixth Annual Meeting of the American Medical Association, held at New York, May*, 1853.

Since the organization of the association in 1847, no meeting has been looked forward to with more anxious solicitude than the one under consideration. A respectable medical journal had stated that it would decide the question, whether another meeting would ever be held, and though but few, it is believed, agreed with this declaration, many thought, and still think, that the action of this meeting was to determine the question, whether the American Medical Association was to become a permanent institution of the country, or was to linger for a few years longer and then cease to exist.

When the present anomalous plan of organization was made public, there was one general burst of disappointment; it was not what was wanted, not what was expected; the profession was distracted, disorganized, and the title of Doctor almost a a by-word and reproach. The degree of M. D. had ceased to be a recommendation, and many of the worst enemies of the profession added M. D. to their names. Among the medical schools there was neither uniformity of requirement, nor harmony of action. Many required three years' study, some two, and some no definite period. Most of them requirec two courses of lectures, some but one. In some schools the lecture term continued but twelve weeks, some sixteen, and in some five months. What was wanted and expected was a plan of organization which would observe a line of demarkation between those who were willing to unite in sustaining our time-honored profession and those who were laboring for its destruction; to produce a united effort for its improvement, and, as subsidiary to this, to establish some uniform standard of requirement for the degree of Doctor of Medicine. In each of these particulars the present organization entirely failed; no provision was made for separating the honest practitioner from the empiric; no bond of union; no standard of requirement for the Doctorate. To the general astonishment of the profession, the fundamental objects of the convention seemed to have been ignored by the committee; so strong were the feelings of dissatisfaction even in the convention, that the constitution was scarce adopted before propositions were made for its amendment. The necessity for an alteration became every year more apparent, until at the fourth meeting held at Charleston, a committee, embracing some of the ablest members, was appointed to report on the subject of amendment to the constitution. At the next meeting at the fifth annual meeting, which was held at Richmond, two separate reports were presented, both recommending amendments; after some discussion the reports were both referred to a select committee; this committee made a report, which, after a full discussion and some slight alteration, was adopted and referred over,—as required by the constitution—to the next meeting. It was these proposed amendments to the organic law of the association, which gave particular interest to the recent meeting, and constituted by far the most important business before the association; it indeed influenced many members in voting to meet at New York instead of St. Louis, as was recommended by the committee. Subsequent to this action, two of the most important state societies represented in the association, had instructed their delegates to vote for the proposed amendments. In order that ample time might be given for discussion, a motion was made on the second day of the meeting to suspend the order of business in order to consider the proposed amendments of the constitution, but it was voted down. On the third day, when all the regular business had been disposed of, and much time wasted in discussing points which were embraced in the proposed amendments, a second motion was made to lay the pending business on the table to take them up, but with the same results. When too late to consider them they were finally taken up, but before the chairman had finished reading the amendments proposed, a motion for indefinite postponement was made and carried. A more gross act of discourtesy to the two preceding meetings and their committees could scarce be conceived. It also shows, what is evident from reading the transactions, that though purporting to be the same association, one meeting has no influence over the action of another; the same subjects are discussed year after year; perhaps some sage resolves or recommendations are passed, and there the matter ends until the next meeting, when the same farce is re-enacted. The writer has not the vanity to suppose that any thing he can offer will save the association from the fate which awaits it. He has been a close observer of its operation since the first proposition in the State Medical Society of New York; is not connected with any clique; was not a member of any committee for forming or amending the constitution, and has no interest except the general interest of the profession. It is, however, due to the members of the medical profession, that the true cause of failure should be known, and that the profession, as a body, should not be held responsible for what they could not control.

If we look only at the constitution, it would be difficult, if not impossible, to say what constituted the American Medical Association, and it is only by looking at the action of the convention that we learn this important fact; here we find the following resolution:

"Be it resolved in behalf of the medical profession of the United States, that the *members of the medical convention*, held in *Philadelphia in May*, 1847, and all others who, in pursuit of the objects above-mentioned, are to unite with or succeed them, *constitute a National Medical Association*, and for the organization and management of the same they adopt the following regulations—*i. e.* instead of the medical profession in the United States they *constitute themselves* and *their successors* into an association. The portion of the constitution which it was proposed to alter is embraced in the second section, as follows:

I. *Title of the Association.*

The institution shall be known and distinguished by the name and title of The American Medical Association.

II. *Members.*

The members of this institution shall collectively represent and have cognizance of the common interests of the medical profession in every part of the United States, and shall hold their appointment to membership either as delegates from local institutions, as members by invitation, or as permanent members.

The delegates shall receive their appointments from permanently organized medical societies, medical colleges, hospitals, lunatic asylums, and *other permanently organized medical institutions,* of good standing, in the United States; each delegate shall hold his appointment one year and until another is appointed to succeed him, and shall participate in all the business and affairs of the association. Each local society shall have the privilege of sending to the association one delegate for every ten of its regular resident members, and one for every additional fraction of more than half this number.

The faculty of every regularly constituted medical college, or chartered school of medicine, shall have the privilege of sending two delegates. The professional staff of every chartered or municipal hospital containing a hundred inmates or more, shall have the privilege of sending two delegates, and every other permanently organized *medical institution, of good standing,* shall have the privilege of sending one delegate.

The members, by invitation, shall consist of practitioners of reputable standing, from sections of the United States not otherwise represented at the meeting. They shall receive their appointment by invitation of the meeting after an introduction from any of the members present, or from any of the absent permanent members. They shall hold their connection with the association until the close of the annual session at which they are received, and shall be entitled to participate in all its affairs as in the case of delegates.

The permanent members shall consist of all those who have served in the capacity of delegates, and of such other members as may receive the appointment by unanimous vote.

Permanent members shall at all times be entitled to attend the meetings and participate in the affairs of the association, so long as they continue to conform to its regulations, *but without the right of voting;* and when not in attendance they shall be authorized to grant letters of introduction to reputable practitioners of medicine residing in their vicinity, who may wish to participate in the business of the meeting as provided for members by invitation.

Every member elect, prior to the permanent organization of the annual meeting or before voting on any question after the meeting has been organized, must sign these regulations, inscribing his name and address in full, specifying in what capacity he attended, and, if a delegate, the title of the institution from which he has received his appointment."

Instead of the above it was proposed to substitute the following:

"Art. I.— *Title of the Association.*

This institution shall be known and distinguished by the name and title of The American Medical Association. It shall be composed of all the members of the medical profession of good standing, who acknowledge fealty and adhere to the code of ethics adopted by this association; and its business shall be conducted by its delegates or representatives, who shall be appointed annually in the manner prescribed in this constitution."

Strike out the whole of Article II, referring to members, and insert the following:

"Art. II.— *Of Delegates.*

§ 1. The delegates to the meetings of The American Medical Association shall collectively represent and have cognizance of the common interests of the medical profession in every part of the United States and shall hold their appointment from county, state, and regularly chartred medical societies; from chartered medical colleges, hospitals, and permanent voluntary medical associations in good standing with the profession. Delegates may also be received from the medical staff of the United States army and navy.

§ 2. Each delegate shall hold his appointment for one year and until another is appointed to succeed him, and he shall be entitled to participate in all the business affairs of the association.

§ 3. The county, district, and voluntary medical societies shall have the privilege of sending to the association one delegate for every two of its resident members .and one more for every additional fraction of more than one half this number.

§ 4. Every state society shall have the privilege of sending four delegates, and in those states where county and district societies are not generally organized, in lieu of the privilege of sending four delegates, it shall be entitled to send one delegate for every ten of its regular members, and one more for every additional fraction of more than half this number.

§ 5. No medical society shall have the privilege of representation which does not require of its members an observance of the code of ethics of this association.

§ 6. The faculty of every chartered medical college, acknowledging its fealty to the code of ethics of this association, shall have the privilege of sending one delegate to represent it in the association; *Provided*, that said faculty consists of not less than six professors, and gives one course of instruction annually of not less than sixteen weeks, on anatomy, materia medica theory and practice of medicine, theory and practice of surgery, midwifery, and chemistry; and provided, also, that the said faculty requires of its candidates for matriculation: 1. That they shall be twenty-one years of age; 2. That they shall have studied three entire years, two of which must have been with some respectable practitioner; 3. That they shall have attended two full courses of lectures, (not, however, to be embraced in the same year,) and one of which must have been in the institution granting the diploma, and also where the students are required to continue their attendance on the lectures to the close of the session; and 4. That they shall show by examination that they are qualified to practice medicine.

§ 7. Admits delegates from the University of Virginia, though it does not conform to the above regulations.

§ 8. That hospitals in good standing, with accommodations for 100 patients, shall be entitled to one delegate.

§ 9. Admits delegates, four each, from the army and navy, and points out the mode of appointment.

§ 10. No delegate shall be registered on the books of the association as representing more than one constituency.

§ 11. Requires every delegate to sign the constitution, &c.

It will at once be seen that these proposed amendments change the whole organization, and make the association, in fact, a representative institution instead of the present anomaly. It draws the line of demarkation between the regular and irregular practitioner; requires from the former a pledge to conform to the system of medical ethics, and thus to sustain the dignity of the profession; makes the delegate his representative, and confers upon him all the honors, the rights and privileges of a member of the American Medical Association, and thus enlists his interest and pride in sustaining it. At the same time, it defines the duties and requirements of the medical schools, and insists that they should require of their pupils a certain standard of acquirement which the present constitution entirely omits. The term, *every other permanently organized institution of good standing*, is so obscure and indefinite that we have already seen the officers differing as to its meaning, and bitterness of feeling introduced on that account. It is unnecessary to draw further comparison; the members of the medical profession can judge for themselves. It is believed that the following propositions, (if they are not self-evident,) are capable of demonstration:

1. That the present organization of the American Medical Association, is not what was demanded by the wants of, and expected by, the profession.

2. That though it has proved valuable in some respects, it has, after an experiment of six years most signally failed in the great objects of its organization.

3. That it has failed to enlist the interest and sympathy of the great body of the profession, and without this it cannot be permanent.

The great object of the association was to elevate the medical profession; but this is impossible until it is determined who constitute the profession. To attempt to elevate the host of empirics who infest community under the name of Thompsonian, botanic and homœopathic doctors, I presume, was never contemplated, and yet the present organization points out no mode of discrimination.

2d. Has the association succeeded in accomplishing the object of elevating the profession? If so, the writer has been unable to discover it. Is there more union, or harmony, among its members — more esprit du corps? Is medical education any more thorough? Are the requirements for the Doctorate any higher?

Doct. W. Hooker stated to the association cases where students had received the degree, one after one year's study, several after eighteen months, and some after two years. Was it any worse six years sincc? And yet these institutions are represented in the association. It is admitted that some of the reports and dissertations published are valuable; that the meetings have usually been pleasant and agreeable. So far the association has been useful. But this will not insure its continuance.

3d. It has failed to enlist the sympathy and interest of the great body of the profession. The population of the United States is about 23,000,000; allowing one regular physician for every thousand — which is a small proportion — you have 23,000 as the number of medical men.

According to the Treasurer's report, 500 copies of the Transactions of the last year were sold, or one in forty-six of the profession. It should, however, be remembered, that every member present at the meeting is expected, as a matter of course, to purchase a copy; there were present at Richmond, 300; deduct these and it leaves but 200 out of those not present at the meeting who take sufficient interest in the proceedings to procure a copy of its Transactions. Out of the fifty-nine counties in the state of New York, but six were represented at Richmond, and even at the recent meeting, when brought to their own doors, some of the largest county societies in the state were not represented. Hitherto three-fourths of the delegates in attendance have been from the large cities and the schools. Even when delegates are appointed by the county societies, the question is not asked, whom shall we send to represent the society, but who wants to go? and if no one volunteers, no delegate is sent. They do not feel that they are under any obligations to send, or assume any responsibility as to the action of the delegate when sent, or that of the association. In fact, more than nine-tenths of the profession in the United States care nothing about the association. If the association means to be really what its name imports, it should embrace within its folds every man of standing and education who feels a pride in his profession, and is willing to unite in an effort for its advancement. Membership should be something more than a name: it should be prima facie evidence of his honorable standing in the profession, and an introduction to the confidence and fellowship of his medical brethren.

An impression has been created, and most industriously promulgated, that the proposed amendments proceeded from hostility to the medical schools, and this was encouraged by the unfortunate statement of the chairman of the committee, that the most important alteration was the reduction of the number of delegates from the schools and hospitals; whereas, in fact, many who voted for the amendments were as much opposed to such reduction as any member of the association, and only yielded to secure what they considered far more important to the permanency and usefulness of the association.

